# A Pathway to Assess Genetic Variation of Wheat Germplasm by Multidimensional Traits with Digital Images

**DOI:** 10.34133/plantphenomics.0119

**Published:** 2023-11-22

**Authors:** Tingting Wu, Peng Shen, Jianlong Dai, Yuntao Ma, Yi Feng

**Affiliations:** ^1^ College of Mechanical and Electronic Engineering, Northwest A&F University, Yangling, Shaanxi 712100, China.; ^2^ Key Laboratory of Agricultural Internet of Things, Ministry of Agriculture and Rural Affairs, Yangling, Shaanxi 712100, China.; ^3^ College of Information Engineering, Northwest A&F University, Yangling, Shaanxi 712100, China.; ^4^ College of Land Science and Technology, China Agricultural University, Beijing 100091, China.; ^5^ College of Agronomy, Northwest A&F University, Yangling, Shaanxi 712100, China.

## Abstract

In this paper, a new pathway was proposed to assess the germplasm genetic variation by multidimensional traits of wheat seeds generated from digital images. A machine vision platform was first established to reconstruct wheat germplasm 3D model from omnidirectional image sequences of wheat seeds. Then, multidimensional traits were conducted from the wheat germplasm 3D model, including seed length, width, thickness, surface area, volume, maximum projection area, roundness, and 2 new defined traits called cardioid-derived area and the index of adjustment (J index). To assess genetic variation of wheat germplasm, phenotypic coefficients of variation (PCVs), analysis of variance (ANOVA), clustering, and the defined genetic variation factor (GVF) were calculated using the extracted morphological traits of 15 wheat accessions comprising 13 offspring and 2 parents. The measurement accuracy of 3D reconstruction model is demonstrated by the correlation coefficient (*R*) and root mean square errors (RMSEs). Results of PCVs among all the traits show importance of multidimensional traits, as seed volume (22.4%), cardioid-derived area (16.97%), and maximum projection area (14.67%). ANOVA shows a highly significance difference among all accessions. The results of GVF innovatively reflect the connection between genotypic variance and phenotypic traits from parents to offspring. Our results confirmed that extracting multidimensional traits from digital images is a promising high-throughput and cost-efficient pathway that can be included as a valuable approach in genetic variation assessment, and it can provide useful information for genetic improvement, preservation, and evaluation of wheat germplasm.

## Introduction

Germplasm resources represent a critical foundation for crop breeding [1,2]. The assessment of genetic variation in crop germplasm has long been a significant topic in agricultural research [3–5]. Wheat, as one of the world’s principal cereal crops, holds a pivotal role in agricultural breeding. Evaluating the genetic variation of wheat germplasm offers invaluable insights for agricultural researchers, aiding them in better understanding and harnessing these resources.

The genetic variation in crop germplasm is shaped by both natural genetic drift and human-directed selection, endowing crops with the capacity to adapt to diverse environmental conditions [6,7]. Phenomics, as a potent research tool, delves deeply into and harnesses these genetic variations, paving the way for more efficacious precision breeding [8]. This domain typically involves the utilization of an array of sensors to capture phenotypic data, followed by phenotypic analysis. While it presents a challenging research landscape, it is also replete with opportunities. Through the lens of phenomics, we can discern the intricate relationships between crop traits and genetic factors with heightened precision [9].

Phenomics has made significant strides in the study of genetic variation in crop germplasm in recent years. He et al. [10] delved into traits such as thousand-grain weight, grain length, and grain width in germplasm and elucidated key genes influencing millet yield attributes. Varshney et al. [11] constructed a genetic variation map from a global collection of 3,366 chickpea germplasms, unveiling superior haplotypes conferring climate adaptability in chickpeas. Chen et al. [12] harnessed natural genetic variations in a leguminous plant to identify genes modulating seed traits and conducted a genome-wide association study on 32 seed-related traits, focusing on seed size and composition. Despite the laudable achievements from these and numerous other studies [13–18] on germplasm genetic variation, challenges such as the intensive labor, extended duration, and potential subjectivity in phenotypic data acquisition persist. To cater to rapid and accurate evaluations of germplasm genetic variation, there is a pressing need to explore novel methodologies that enhance the efficiency and precision of research into wheat germplasm variation.

With the evolution of digital imaging methodologies, phenomics has entered a new phase in the study of genetic variation. Image analysis can be considered a measurement tool, where automated image processing techniques allow for higher throughput, reliability, and repeatability at various scales, ranging from microscopic to field levels. Typically, 2-dimensional (2D) images are used to calculate parameters such as width, length, and area [19–22], while other mathematical operators are employed to derive more intricate morphological traits [23]. For cereal crops, measuring detailed 3D morphological information (e.g., volume, surface area, and furrow) remains a formidable task, even though they are associated with nutrient translocation capabilities and crop growth processes. Pinpointing these pivotal phenotypes necessitates intricate measurement techniques and data processing methods. While digital imaging offers a rapid, high-throughput, and cost-effective means for seed genetic variation studies, its primary focus is on 2D traits, limiting the detectable traits for minuscule seeds. The limitations of image processing on multidimensional traits considerably constrain its applicability in wheat germplasm genetic variation research.

3D reconstruction technology, with its capability to capture an expansive range of multidimensional traits, has heralded a new revolution in agricultural breeding research. Methods based on active light, employing tools like x-ray computed tomography (CT) and laser scanners, boast high precision but come at a greater cost and complexity. Studies by Li [24], Huang et al. [25], and Yang and colleagues [26,27] have adeptly harnessed these tools for examining rice grain features, while Zhu et al. [28] demonstrated the efficacy of oblique photography as a more efficient alternative. However, the steep costs and intricate operation of these devices remain inherent challenges. Image-based reconstruction techniques such as structure from motion (SFM) [29] and multi-view stereo (MVS) [30,31] are promising, but their deployment on seeds with uniform textures, like wheat seeds, poses challenges. An innovative approach introduced by Roussel et al. [32] utilized voxel space carving with digital images for seed reconstruction, unveiling several multidimensional features. The author hoped that this research would pique increased interest in voxel reconstruction methodologies. Nevertheless, seeds reconstructed using this technique exhibited discernible cut marks, and there was a lack of subsequent analysis on the role of the measured traits in genetic variation. These prior investigations illuminate the potential and the challenges of 3D reconstruction in wheat seed phenotypic trait research, especially when digital images are the medium for 3D reconstruction. To fully capitalize on this approach and navigate its limitations, it is imperative to further refine 3D reconstruction techniques and delve deeper into the implications of measuring multidimensional seed traits.

This research presents a pathway for assessing genetic variation in wheat germplasm using multidimensional traits derived from digital images. The specific objectives were to (a) construct a machine vision platform to acquire images of wheat seeds; (b) perform a 3D reconstruction of wheat seeds within the modeling of the rotational axis, using a voxel reconstruction method that requires only one calibration for high-precision 3D reconstruction; (c) extract multidimensional phenotypic traits from the reconstructed model; and (d) evaluate genetic variation using phenotypic coefficients of variation (PCVs), analysis of variance (ANOVA), and the defined genetic variation factor (GVF). This research aims to provide useful information for the genetic improvement, preservation, and evaluation of wheat germplasm.

## Materials and Methods

### Experimental design

**Fig. 1.**
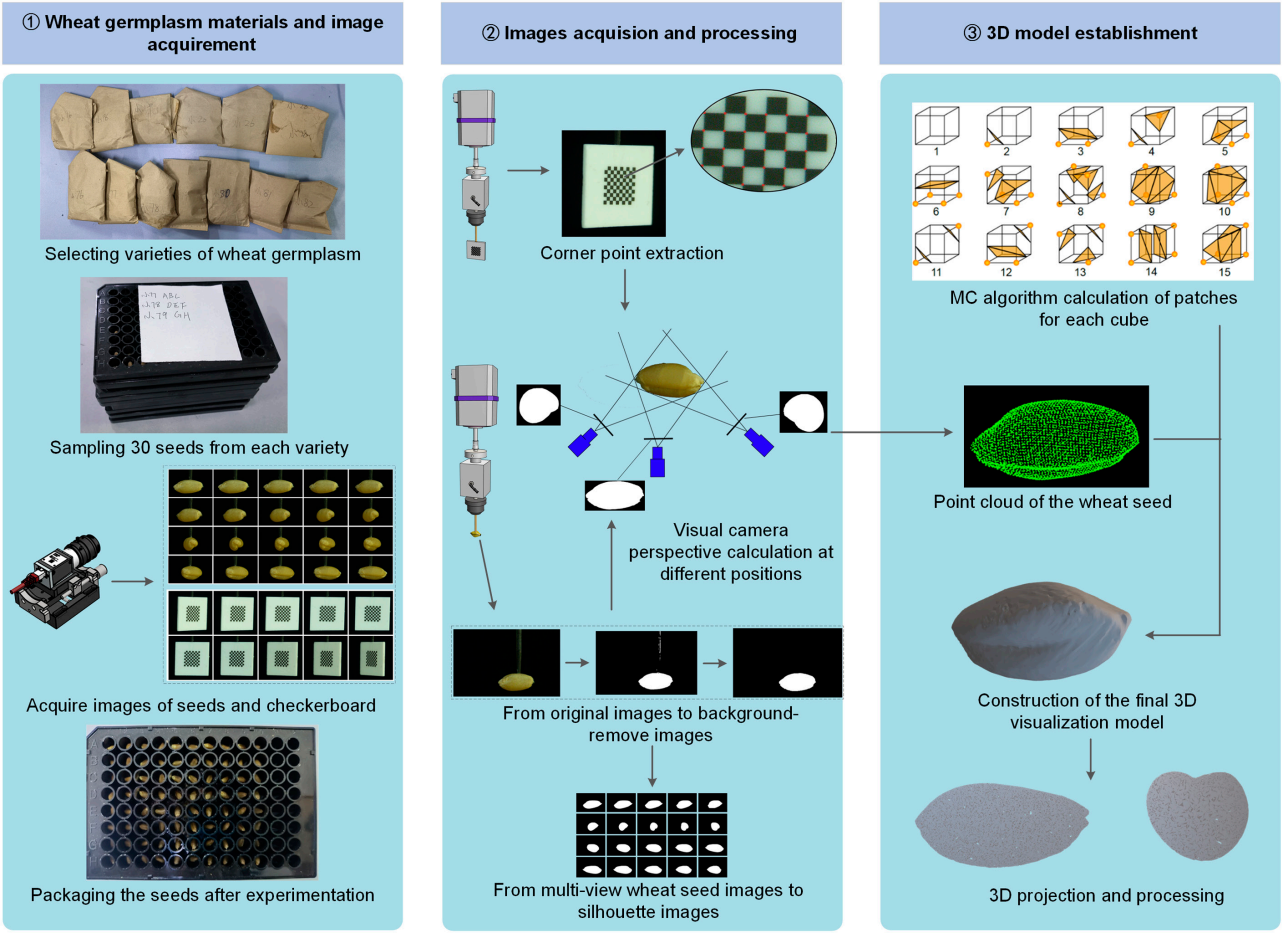
General schematic diagram of the 3D construction. The figure presents the 3D model reconstruction process in order of the experiment, which is divided into 3 parts.

The evaluation of genetic variation in wheat germplasm was comprehensively assessed using a combination of phenotypic coefficient of variation (PCV), variance analysis (ANOVA), clustering, and a newly defined GVF. The validity of these results was contingent upon the accuracy of the extracted phenotypic traits. To obtain multidimensional phenotypic traits of wheat seeds, a phenotyping system utilizing omnidirectional images was developed, as shown in Fig. 1. This system consisted of 2 steps:

• Construction of a machine vision platform: Composing with a nozzle to attach wheat seeds and a checkerboard, a high-precision rotation step motor, and a control system to capture images, allowing us to obtain images of the wheat seeds and checkerboard from different angles.
• Establishment of a 3D seed model: Composing of 3D model reconstruction of wheat seed, modeling of the rotational axis, and parameter traits extracted from the model.

### Materials and image acquirement

Fifteen wheat seed varieties were selected for the assessment of genetic variation. The chosen varieties comprise 2 parental strains, Xinmai 26 and Xinong 294, and 13 offspring varieties derived from them. The parental strains, cultivated over several years, are excellent varieties that cover a broad spectrum of seed traits. Their hybrid offspring bear significance for the study of genetic variation. The offspring varieties are divided into 2 batches. The first batch contains 6 varieties derived from a crossbreed between Xinmai 26 and Xinong 294. The second batch consists of 7 varieties, which were obtained by first crossbreeding Xinong 20 and 02Ta, followed by a crossbreed with Xinong 585. A total of 450 wheat seeds, with 30 seeds randomly selected from each variety, were subjected to the experiment. The detailed information of the varieties is shown in Extended Table 1.

The assessment of genetic variation was based solely on digital images. Prior to the experiment, 8 to 12 checkerboard images were acquired and utilized to calibrate the camera poses in the system. A 9 × 9 checkerboard was utilized with a corner-to-corner distance of 0.5 mm, and the camera pose calibration was performed only once. During the experiment, omnidirectional image sequences of 450 wheat seeds were acquired at 9-degree intervals, totaling 40 images per seed. The acquired calibrated images and 450 × 40 images of the wheat seeds were analyzed and processed to assess the genetic variation in wheat germplasm. The data from these images served as the sole input for the entire method.

### Construction of a machine vision platform

**Fig. 2.**
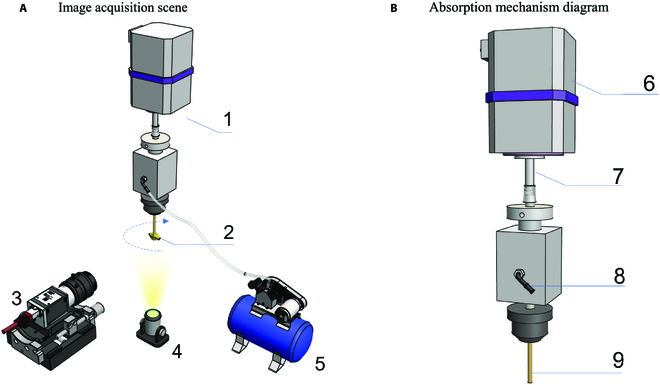
(A and B) The machine vision platform for digital images of wheat seed and checkerboard. The entire platform is composed of 5 parts: (1) absorption mechanism (absorbs the sample and rotates it 360 degrees), (2) sample (wheat seed or checkerboard), (3) CCD camera (captures images throughout the rotation), (4) ring light (provides consistent lighting), and (5) vacuum pump (creates suction, enabling the sample to be absorbed). The absorption mechanism consists of 4 parts: (6) stepper motor (provides torque, allowing the sample to rotate at specific angles), (7) connecting shaft (links the motor and the suction nozzle), (8) air tube (connects to the vacuum pump, for creating suction so the wheat seed can be absorbed), and (9) suction nozzle (used for absorbing the wheat seed).

To obtain a complete sequence of wheat seed images, a machine vision platform has been constructed that incorporates a precision rotating mechanism and a clever suction device, as shown in Fig. 2. The platform consists of several key components, including an industrial charge coupled device (CCD) camera, a suction nozzle, a connecting shaft, a vacuum pump, and a stepper motor. The industrial camera is composed of a microscope camera (model KUY NICE-CM1000, 4,912 × 3,684 pixels, Shenzhen Kuy Nice Microscope Co. Ltd., China) and a fixed-focus industrial lens (model WP-2M2514-C, 25 mm focal length, 20 × 16.8 mm field of view, Shenzhen Work Power Technology Co. Ltd., China), which is fixed by the camera bracket to capture high-resolution images of the checkerboard or wheat seeds. The camera faces the area below the suction nozzle of the device to ensure that the object to be photographed is completely within the camera’s view. The suction nozzle is directly inserted into the nozzle seat and attached to the connecting shaft, with a small vacuum pump connected to the side of the seat. The top of the connecting shaft is connected to the stepper motor.

Wheat seeds or a checkerboard were stably adsorbed on the suction nozzle after activating the air pump. The stepper motor drove the suction nozzle to rotate in 9° increments, collecting a total of 40 omnidirectional image sequences of the wheat seed at different angles. The contact surface between the suction nozzle and wheat seed was minimal, ensuring the completeness of the captured images. Before capturing wheat seed images, images of a checkerboard were also obtained for calculating the virtual angle of views. It was ensured that the interval between each rotation was 9° or 4.5°, and 9 checkerboard image sequences were collected. Accordingly, software was designed to automate this process. After fixing the machine vision platform, it was only necessary to capture images once with the checkerboard attached to the suction. Then, the number was input and the first wheat seed was placed onto the machine vision platform. Once it was finished, the next one was placed on. These were all the operations that needed to be performed manually. Each time the nozzle rotated, the CCD camera took a picture and saved it, which was all executed automatically by the software.

### Establishment of a 3D seed model

In order to study the genetic variation more thoroughly in wheat germplasm, it is necessary to comprehensively and multidimensionally characterize the traits of wheat seeds. However, due to the small size of wheat seeds, manual nondestructive measurement methods and image processing-based methods are prone to large errors. Therefore, this study proposes a method to build 3D models of wheat seeds considering the machine vision platform. The method is based on IBVH [33], which constructs 3D models from multiple digital images. Also, an axis-based way was proposed to calculate the virtual views, ensuring the high precision of the 3D model. Leveraging the wheat seed model, multidimensional phenotypic traits can be extracted.

MATLAB R2018b is used to calculate the virtual view, C++ OpenCV library is used to process digital images, and C++ PCL (Point Cloud Library) [34] and VTK (Visualization Toolkit) are used to build, display 3D models, and extract phenotypic traits. The C++ development software is Microsoft Visual Studio 2017. In addition, MeshLab software is used to facilitate the viewing and display of the point cloud results.

#### 3D model reconstruction of wheat seed

A method was proposed for building 3D models of wheat seeds using a machine vision platform. The 3D reconstruction process involved 3 main steps: image processing, projection matrix calculation, and face slice reconstruction.

First, images obtained from the vision platform were processed using image binarization, background removal, and silhouette extraction to get high-quality contour images of wheat seeds from 40 different viewpoints. Background removal is a crucial step in the contour extraction process. A method was utilized that segments the suction nozzle background by identifying the horizontal intersection line between the suction nozzle and the wheat seeds when the total number of white pixel points in the same row changes more than a certain percentage compared to the previous view. Additionally, an algorithm based on a localized Radon transform [35] was employed to extract the 81 corner points of the checkerboard image under each viewpoint. This information is then used to obtain the spatial position information of the corner points in the set world coordinate system under 9 different camera views.

Second, the calculation of the projection matrix, as shown in Eq. 1, was performed. This involves determining the camera’s internal and external parameters using the classical linear camera model (the pinhole model) for the conversion of the world coordinate system to the pixel coordinate system. The camera internal parameters are represented by the matrix K, which includes 5 parameters: *f* (effective focal length), *dx* and *dy* (object size of each pixel in the image horizontal and vertical directions), *α_u_* and *α_v_* (image horizontal and vertical scaling factors), and *s_uv_* (distortion factor). The rotation matrix *R* and the translation vector *T* are used to describe the rigid body transformation relationship between the camera coordinate system and the world coordinate system, forming a 4 × 4 matrix to determine the camera’s pose, which are the external parameters of the camera. These internal and external parameters are then combined to form a 3 × 4 projection matrix *P*, which represents the geometric relationship between a 3D point in space and its projection to a 2D point in the imaging plane. A calibration method based on the rotation axis model was proposed to calculate the camera’s external parameters.

zCuv1=zC1dx0u001dyv0001xy1=zC1dx0u001dyv0001f0000f000010xcyczc1

=αusuvu000αvv000010RT0T1xwywzw1=K∙RT0T1xwywzw1=Pxwywzw1 (1)

Finally, the 3D model of wheat seed was reconstructed by applying the face slice reconstruction method. The projection matrix calculates the projection of voxel points in space under each viewpoint, which enables the classification of spatial voxel points into 3 states: points on the seed 3D model, points inside the seed 3D model, and points outside the seed 3D model. The MC (Marching Cubes) algorithm [36] was utilized to reconstruct the 3D faceted model of wheat seeds based on the states of voxel points in space.

#### Modeling of the rotational axis

Based on the constructed rotational visual platform, an accurate method for modeling the rotational axis has been designed. In the whole process of virtual view construction, the calculation of camera internal parameters and the establishment of the final projection matrix are the basic steps in the 3D reconstruction process. In the middle, the calculation method of the rotation axis is the key of the experiment, and its accuracy directly affects the accuracy of the reconstruction results. According to the spatial arc coordinates of each corner point in the camera coordinate system *O_C_X_C_Y_C_Z_C_* extracted, the circle centers of space are solved. These circle centers are located on different positions of the rotation axis. The 3D spatial straight line where the set of circle center points is located is fitted, which is the requested rotation axis. In order to obtain more robust calculation results, multiple corner points are used to participate in the estimation.

The corner points *P_i_* were set to constitute the data set *P*, and the circle center *O* and radius *R* were obtained by fitting the spatial sphere, that is, to find

$$\min_{O,R}\sum_{i}\left|\left\|O-P_{i}\right\|-R\right|$$ (2)

Based on the linear regression of principal component analysis to fit the rotation axis, the centers of these circles ***O***_***i***_, *i* = 1, …*N*. The average value of *N* is:

$$m=\frac{1}{N}\sum_{i=1}^{N}O_{i}$$ (3)

Finally, calculate the covariance matrix Λ for the centers of these circles *O_i_*, as shown in Eq. 4.

$$\Lambda =\sum_{i=1}^{N}\left(O_{i}-m\right){\left(O_{i}-m\right)}^{T}$$ (4)

The eigenvector *n* corresponding to the largest eigenvalue of *λ* is obtained, namely, the rotation axis.

#### Parameter extracted from the model of the seed

By referring to the related research on wheat seed phenotypic identification [25], we determined 9 wheat phenotypic traits based on 3D reconstruction data, as shown in Table 1. Among them, length, width, and thickness are the maximum distances in the 3 axial directions of the reconstructed contour envelope, respectively. The surface area is obtained by combining a series of grid-based triangular patches based on the boundary voxels and the iso-surface. By slicing the grain contour boundary at equal intervals along the longitudinal direction and sufficiently small intervals, the volume can be calculated by integrating and accumulating each slice. The projection area is determined by the slice with the maximum area. Additionally, 2 new phenotypic traits were proposed to respond to the ventral groove trait of wheat seeds: the cardioid-derived area and J index. Since the groove is a relatively obvious trait but difficult to analyze, the groove face was oriented upward and projected onto a heart-shaped curve. The resulting area, called the cardioid-derived area, provides a more accurate representation of the groove than traditional methods. To normalize measurements for seeds of different sizes, J index was calculated by dividing the cardioid-derived area by the square area of the seed. These 2 traits were designed to offer more detailed information about the shape and size of wheat seeds.

**Table 1.** Phenotypic traits of wheat seed

Phenotypic traits	Acronym	Computational formulas
Seed length	*L*	*L* = *x_max_* − *x_min_*
Seed width	*W*	*W* = *y_max_* − *y_min_*
Seed thickness	*D*	*D* = *z_max_* − *z_min_*
Surface area	*S*	$S=\sum_{i=1}^{n}\sqrt{l\left(l-a_{i}\right)\left(l-b_{i}\right)\left(l-c_{i}\right)}$
Volume	*V*	V = [(*z_max_* − *z_min_*)/*n*] $\sum_{i=0}^{n-1}\frac{A_{i}+A_{i+1}}{2}$
Projection area	*A*	*A* = max (*A_i_*), *i* = 0, 1, …*n* − 1
Roundness	*R*	$R=\sum_{j=0}^{m-1}\sqrt{{\left(x_{j}-x_{j+1}\right)}^{2}+{\left(y_{j}-y_{j+1}\right)}^{2}}$
Cardioid area	*A_C_*	$A_{C}=\max\left(A_{i}^{W\times D}\right)$
J index	*J*	*J* = *A_C_*/(*W* × *D*)

Following the extraction of phenotypic traits, a comparison between manual measurements and 3D reconstruction measurements was conducted. The coefficient of determination (*R*^2^) was employed to reflect the regression relationship between manual and 3D reconstruction measurements, while the root mean square error (RMSE) was used to quantify the errors. In the manual measurements, a vernier caliper was utilized to hold the wheat seeds, providing precise length measurements. However, it should be noted that the measurements for width and thickness may exhibit slight deviations at the millimeter scale.

### Assessment of the genetic variation

**Fig. 3.**
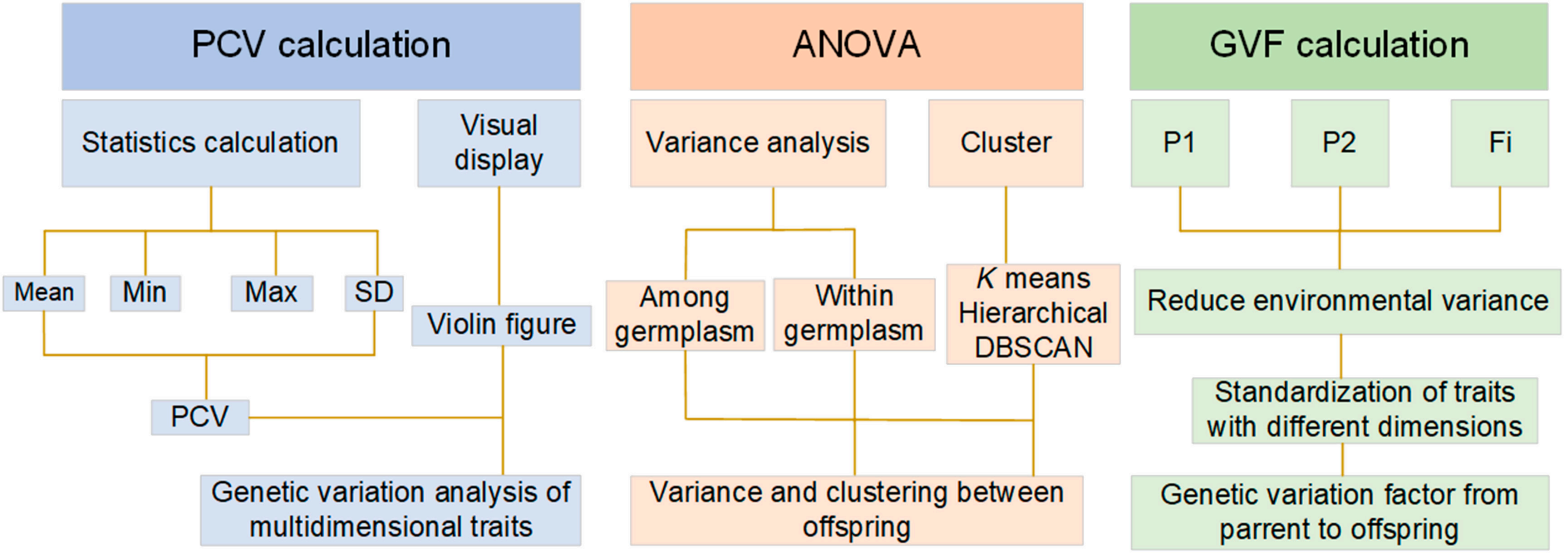
Diagram of assessment of the genetic variation. The assessment methods for genetic variation can be mainly divided into 3 parts in a progressive relationship: PCV calculation, ANOVA (including variance analysis and clustering), and defined GVF calculation.

A 3-step process (PCV calculation, ANOVA, and GVF calculation) was employed to assess the genetic variation in wheat seeds, using the 3D model derived from images taken at different angles, as shown in Fig. 3. Through PCV calculation, the effectiveness of multidimensional traits in expressing genetic diversity in wheat seeds was verified. ANOVA allowed for the analysis of the variance in phenotypic traits between different generations, providing insights into genetic variation in wheat. Finally, in the GVF calculation step, genetic variation was estimated by considering environmental factors that may affect the inheritance process. This study highlights the importance of taking into account environmental factors in the estimation of genetic variation, and the potential of using multidimensional traits to better express genetic diversity in wheat seeds.

**Fig. 4.**
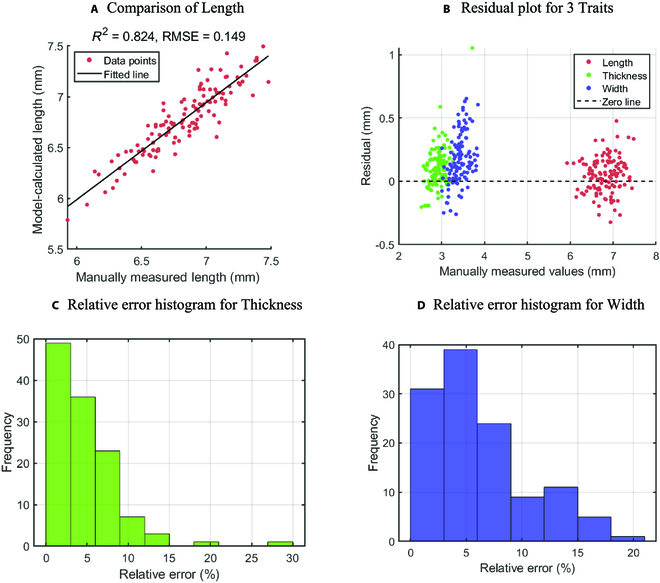
(A to D) Comparison of wheat kernel morphologies between reconstruction and manual measurement.

#### Genetic variation for multidimensional traits

The coefficient of variation (CV) is a useful metric for quantifying data dispersion, controlling for measurement scale and dimension. In the agricultural sector, the use of phenotypic coefficient of variation (PCV), genetic coefficient of variation (GCV), and environmental coefficient of variation (ECV) is commonplace in the assessment of crop character diversity in response to various factors [37,38]. When environmental conditions are controlled, the PCV of each character in parents and offspring can provide insights into the diversity of germplasm for a particular variety, as shown in Eq. 5.

$$\mathrm{PCV}=\frac{\sigma_{P}}{\bar{\delta_{j\left(i\right)}}}$$ (5)

where *δ*_*j*(*i*)_ is the effective value of the *j*th individual of the *i*th germplasm and *σ_P_* is the standard deviation (SD) of phenotypic traits.

To gain a deeper understanding of genetic variation in multidimensional traits, it is imperative to accurately determine the CV (PCV) for each trait within the germplasm. This process involves calculating the SD of the phenotypic traits and then dividing that value by the average effective value of each sample in the germplasm set. Through this methodology, we can assess the degree to which diversity in each germplasm variety is influenced by elements like genetics and environmental factors. A lower PCV indicates that a trait is relatively stable, with only subtle differences between seeds or varieties. Conversely, when PCV is higher, it signifies that there is a notable variance in trait expression. Such traits are of higher value in genetic research since they can be more effectively associated and analyzed. This foundational step is essential for understanding the genetic variability in wheat and other crops.

Statistical measures were calculated for all wheat grain samples from 15 varieties, including the mean, minimum, maximum, SD, and phenotypic CV for nine traits. Following the calculation of SD, PCVs for each trait were computed. These PCVs were then ranked to identify traits exhibiting the greatest variability. Finally, to further analyze the genetic variation of traits based on phenotypic data, violin plots [39] were drawn for the 3 traits with larger variation coefficients. Data for the statistical measures were directly calculated using Excel spreadsheets. Visualization results were generated using MATLAB R2018b software, and the Violinplot-Matlab-master library was employed for creating violin plots.

#### Variance and clustering between different offspring

ANOVA and cluster were utilized to analysis genetic variation between different offspring. The ANOVA analysis was used to assess the variation between 2 groups of progeny, including those produced via binary hybridization between Xinmai 26 and Xinong 294, and those resulting from ternary hybridization between Xinong 2002Ta and Xinong 585. Individual ANOVAs were conducted for both within and across germplasm, evaluating the differences among the 6 variants of the first progeny, as well as comparing both the first and second progeny to the parental plants. Analysis focused on the *F* value, calculated for each ANOVA, and the differences observed between groups.

Subsequently, clustering was employed to classify the data into distinct groups. Three different clustering methods, namely *K* means, hierarchical, and DBSCAN (density-based spatial clustering of applications with noise) were utilized to categorize 390 wheat germplasm into 2 classes. Varieties derived from binary hybridization (Xinmai 26 and Xinong 294) were designated as category 0, while those from ternary hybridization (Xinong 20, 02Ta, and Xinong 585) were classified as category 1. The clustering analysis took place after measuring wheat seed trait parameters using a 3D reconstruction method. The purpose of this analysis was to assess the reliability of the clustering-based variety analysis approach by comparing the outcomes yielded through clustering.

The final results of the ANOVA and clustering are presented in the form of graphs and charts. The ANOVA is conducted using R programming language for data processing, with the ultimate results presented in tabular format. The outcomes of clustering are visualized through scatter plots generated in 3 different ways, accompanied by histograms that display the various evaluation metrics of the clusters. MATLAB R2018b software is employed for the clustering analysis and plotting, incorporating the Statistics and Machine Learning Toolbox.

#### GVF from parent to offspring

The calculation of the PCV among parents and offspring, as well as the difference analysis between varieties of parents and offspring, can reflect the genetic variety to a certain extent. However, the changes of phenotypic traits not only include the value of genetic variation but also environmental variation to a large extent. In fact, the calculation of the former cannot exclude the influence of environment on the phenotypic variation. In order to reduce the interference of environmental error variance as much as possible and evaluate the genetic variation of the traits from parent to offspring, a method of calculating genetic coefficient is proposed.

Calculating the GVF, which reflects genetic diversity from parent to offspring, assists in understanding trait inheritance and species or population genetic makeup. The complexity of this calculation arises from the influence of both genetic and environmental factors on phenotypic traits. Truncation error estimation helps mitigate the impact of environmental variables.

Cumulative mean values of parent varieties are computed, and differences between offspring varieties are determined. The square root of the parent–offspring difference is calculated using the principle of covariance, obtaining the change value of the trait under the response gene’s influence. Dividing the change value by the mathematical expectation value of the parent traits yields the genetic coefficient. This coefficient indicates genetic variation from parent to offspring and the extent to which offspring traits resemble those of the parent. Considering both genetic and environmental factors provides a comprehensive understanding of species or population genetic makeup. Equation 6 presents the genetic coefficient calculation, with *P*_1_ and *P*_2_ denoting parent varieties and *F_i_* referring to the *i*th offspring variety resulting from parental breeding.

$$\mathrm{GVF}=\frac{\sqrt{\left|\frac{1}{N}D\left(P_{1}+P_{2}\right)-D\left(F_{i}\right)\right|}}{E\left(P_{1}+P_{2}\right)}$$ (6)

## Results

### 3D model accuracy and cycle-time efficiency

Fifteen wheat varieties were employed to reconstruct 3D models, comprising 2 parental and 13 offspring lines. A total of 450 seed samples were collected by randomly selecting 30 seeds from each variety. These samples facilitated the successful reconstruction of the wheat grain’s complete 3D shape and extraction of 9 desired phenotypic traits.

The duration was measured across 3 phases of the 3D modeling process: image capturing, 3D modeling, and trait measurement. During image capturing, photographs were taken at intervals of 9 degrees, each followed by a pause of 300 ms to facilitate camera shooting. This phase, which involved manual placement and image capturing of the seeds, took an average of 40 s per seed. 3D reconstruction, facilitated by CUDA on a GTX 1650, allowed the reconstruction of approximately 450 wheat seeds at a voxel resolution of 128 × 128 × 128 within roughly 13 h. During trait measurement, calculation of surface area and volume through meshing required 40 to 60 s per seed, while other traits could be calculated in less than 1 s per seed. Given that these 3 processes can be executed simultaneously on a single computer, the average time for complete trait measurement of wheat seeds was approximately 100 s per seed. The method, which necessitates only a low-cost industrial camera, turntable, and stepper motor, facilitates multidimensional trait measurement of wheat seeds. It presents a significantly more cost-effective solution than active light reconstruction technologies, such as CT and laser scanners, and outperforms CT reconstruction and manual measurement in terms of time efficiency. Further comparison of results can be found in Extended Table 3. Thus, this method offers an effective blend of high throughput and low cost.

To verify the reliability of the experimental results, 120 seed samples from 4 varieties (S77, S78, S79, and S80) underwent analysis. Several manually measurable phenotypic traits were assessed and compared to the model-derived values. Out of the 9 traits, only length, thickness, and width could be easily measured manually. An electronic caliper with a precision of 0.01 mm was employed for these measurements. For wheat grain length, the top and bottom of the seed were clamped, yielding a relatively accurate value. Each seed’s length was measured thrice, with the average value recorded. Given the potential for larger inaccuracies in manual measurements of wheat seed thickness and width, RMSEs are used to reflect measurement discrepancies. The seed surface is uneven and the thickness is nonuniform, necessitating the division of each wheat grain into 3 groups. Each group was measured thrice, with the maximum value selected. The average of these 3 measurements provided the final manual measurement result. To effectively compare manual measurements with 3D model results, a scatter plot was constructed and a line was fitted, as depicted in Fig. 4A.

The RMSE for length, thickness, and width was 0.15, 0.19, and 0.26, respectively, all falling within 0.3, as depicted in Extended Fig. 3. Since measurements of width and thickness can be easily influenced by subjective factors during the measurement process, we employed the residual plot and relative error histogram to capture these measurement errors, as illustrated in Fig. 4B to D. Most measurement points are concentrated within an absolute error of 0.5 mm and a relative error range of 10%. This observation indicates that the measurements derived from the 3D model of the wheat seed are largely consistent with the real values, providing a reasonably accurate representation of the seed’s attributes.

### Genetic variation expressed from multidimensional traits

Table 2 showcases the outcomes of various statistical analyses. The 9 traits are divided into 3 categories: 1D traits (length, thickness, width), 2D traits (maximum projection area, cardioid area), and 3D traits (surface area, volume, roundness, J index). Among these, PCV reveals interesting insights. While there may be significant variations in the volumes of wheat grains, certain traits show more consistent and limited variations. For instance, the J index, despite being a 3D trait, demonstrates limited disparities, suggesting that differences in furrow traits are minimal compared to other traits. In terms of PCV values, the traits are ranked from highest to lowest as follows: volume, cardioid area, projection area, surface area, width, thickness, roundness, length, and J index. The findings from PCV calculations highlight that 2D and 3D traits, notably volume, heart-shaped projection area, and surface area, tend to have larger PCV values. In contrast, the three 1D traits consistently show smaller PCV values.

**Fig. 5.**
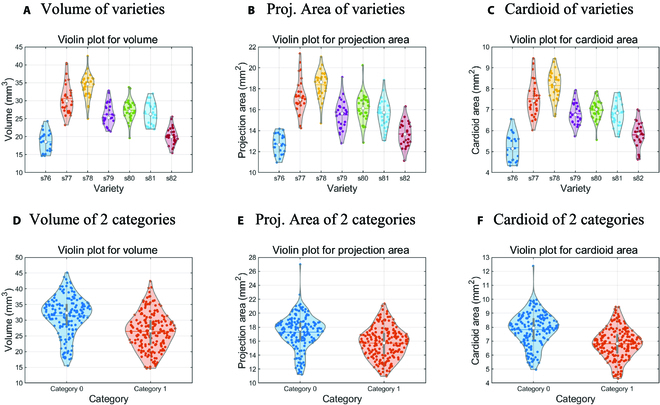
(A to F) Violin plots of the 3 traits with the highest PCV values. Based on the ranking of phenotypic coefficient of variation, the traits with the highest values for volume, projection area, and cardioid area were selected. Violin plots were then generated for these traits for 7 identical progenies and 2 different groups of progenies.

**Table 2.** Statistical characteristics of the quantitative traits

Traits dim	Quantitative traits	Mean	Min	Max	SD	PCV
One- dim	Length	6.67 mm	5.51 mm	7.68 mm	0.44 mm	6.55%
	Thickness	2.83 mm	2.05 mm	3.46 mm	0.24 mm	8.50%
	Width	3.23 mm	2.50 mm	5.83 mm	0.31 mm	9.49%
Two- dim	Proj. area	16.45 mm^2^	10.96 mm^2^	26.98 mm^2^	2.41 mm^2^	14.67%
	Cadioid	7.29 mm^2^	4.33 mm^2^	12.38 mm^2^	1.24 mm^2^	16.97%
Three-dim	Surface	52.36 mm^2^	35.23 mm^2^	73.56 mm^2^	7.54 mm^2^	14.39%
	Roundness	0.61	0.50	0.78	0.04	7.30%
	Volume	28.77 mm^3^	14.65 mm^3^	45.12 mm^3^	6.45 mm^3^	22.44%
	J index	0.79	0.70	0.87	0.03	4.26%

Furthermore, regarding the furrow phenotype of the wheat grain, 2 associated traits have been identified: the heart-shaped area and the heart-shaped derivative index. Among the 9 traits, the heart-shaped area’s PCV ranks second, suggesting its potential significance in genetic variation research, while the J index has the smallest PCV, indicating its stability across different grain varieties.

Three traits with the highest PCV rankings were selected, and the resulting violin plots are presented. As depicted in Fig. 5A to C, comparisons were made within the second category of offspring, revealing differences in the distribution of various varieties within the same batch. Notably, the distributions of the 3 traits for S76 and S82 were relatively small. Agricultural breeding experts may consider excluding these 2 grain varieties from future breeding endeavors. Figure 5D to F display comparisons between the 2 offspring batches. Although the distribution values indicate similarities between the 2 batches, the first batch exhibited slightly larger corresponding traits than the second. Furthermore, the data concentration degree reveals a more concentrated distribution for the second batch. These violin plot results can serve as a preliminary reference for breeding experts.

### Variance and clustering among diverse offspring

ANOVA revealed significant differences in both intraspecific and intermediate traits, with interspecific differences being more substantial than intraspecific differences, as displayed in Table 3. All traits exhibited significant differences, as indicated by a *P* value less than 0.01, which applied to both within and among germplasm. Nevertheless, the extent of these differences varied, as demonstrated by the *F* values. Excluding the 3 traits of length, surface area, and volume, *F* values for the remaining 6 traits were higher among germplasm than within germplasm. Consequently, ANOVA results suggest that variability among different offspring is somewhat greater than within the same offspring. However, these results cannot directly determine the classification of seeds originating from distinct offspring.

**Table 3.** Statistical characteristics of the quantitative traits

Quantitative traits	Mean square			*F* value	
	Among germplasm	Within germplasm	Random errors	Among germplasm	Within germplasm
Length	6.130	4.801	0.180	34.046**	86.092**
Width	2.272	0.663	0.068	33.588**	27.640**
Height	3.671	0.895	0.088	41.571**	11.410**
Surface	2,123.200	1,145.146	55.210	38.457**	56.969**
Volume	1,859.699	834.282	38.411	48.415**	58.223**
Proj. area	213.662	106.953	5.283	40.442**	38.915**
Roundness	0.012	0.007	0.002	5.785**	4.013**
Cardioid	74.348	23.287	1.464	50.789**	31.921**
J index	3.314	0.008	0.001	2,430.353**	12.594**

Note: ** Extremely significant difference (*P* < 0.01).

**Fig. 6.**
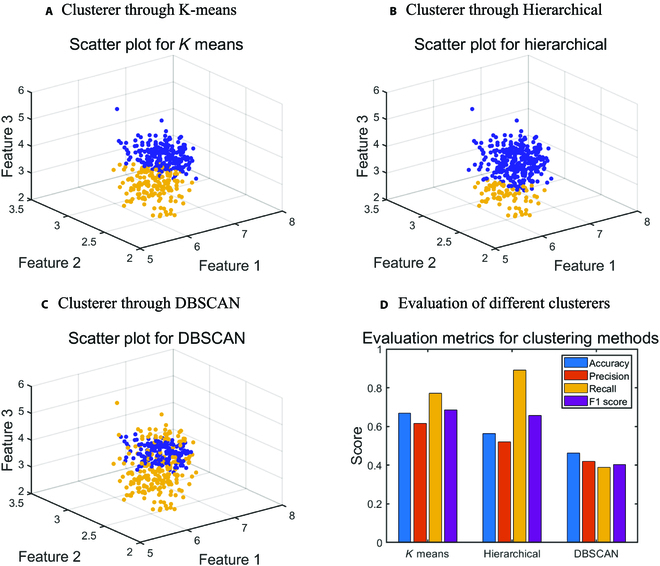
(A to D)Clustering scatter plots and evaluation of 3 different clusterers: *K*-means, hierarchical, and DBSCAN. Both *K*-means and hierarchical approaches exhibit favorable clustering performance.

According to the ANOVA results, various methods can effectively cluster wheat seeds into 2 categories, as illustrated in Fig. 6. It is well established that seed morphological characteristics are subject to considerable random variations due to environmental influences. The ANOVA findings also suggested that variability among germplasm was not substantially greater than within germplasm. As a result, direct classification of categories based on phenotypic traits is not feasible. However, unsupervised clustering can be conducted using phenotypic variation in wheat seed traits. In Fig. 6A to C, it is evident that wheat seeds can be clustered into 2 categories reasonably well. Last, a comparison of the clustering outcomes with actual label values revealed that the precision score of *K*-means clusterer is 70%, as demonstrated in Fig. 6D.

### GVF from parent to offspring

The devised GVF quantitatively reflects genetic changes from parent to offspring. The “Genetic variation expressed from multidimensional traits” and “Variance and clustering among diverse offspring” sections demonstrate that seed phenotypic traits can respond to genetic variation to a certain degree, albeit heavily influenced by environmental factors. GVF calculations aim to minimize the impact of environmental errors. GVF values for 9 traits of the second offspring variety, corresponding to the 2 parents, were computed and are presented in Table 4. It is evident that surface area values significantly decreased in this offspring variety. The 4 traits with the highest GVF values are volume, cardioid area, width, and projection area. GVF results innovatively reveal the relationship between genotypic variance and phenotypic traits from parents to offspring.

**Table 4.** Statistical characteristics of the quantitative traits

Quantitative traits	*F*_1_	*F*_2_	*F*_3_	*F*_4_	*F*_5_	*F*_6_	*F*_7_
Length	2.8%	1.1%	2.3%	1.6%	0.6%	2.8%	3.2%
Width	7.8%	2.3%	4.4%	4.7%	4.4%	3.2%	4.7%
Thickness	1.8%	1.7%	3.1%	3.7%	3.6%	3.7%	5.5%
Surface	6.1%	5.9%	3.4%	3.1%	2.5%	4.4%	4.2%
Volume	8.2%	11.2%	6.6%	2.5%	5.6%	4.2%	5.4%
Proj. area	2.1%	7.4%	3.0%	4.6%	4.2%	3.7%	5.7%
Roundness	3.1%	2.3%	6.5%	6.8%	1.7%	3.7%	4.3%
Cardioid	8.2%	7.8%	2.4%	6.3%	7.0%	4.3%	3.0%
J index	1.8%	0.9%	0.4%	3.0%	4.0%	1.4%	3.7%
Average	4.6%	4.5%	3.6%	4.0%	3.7%	3.5%	4.4%

the feasibility of the 3D reconstructionNote: *F*_1_ to *F*_7_ represent 7 different varieties of the studied plant. The percentages indicate the coefficient of genetic variation for each quantitative trait in each variety.

In the “Genetic variation expressed from multidimensional traits” section, the coefficient of phenotypic variation was calculated, and it was hypothesized that multidimensionality might better reflect genetic variation. However, since phenotypic variation encompasses both genetic variation and environmental error, minor environmental errors may be amplified in multidimensional traits. Consequently, it is not possible to directly confirm that top-ranked traits possess larger genetic variations through the coefficient of phenotypic variation. Nevertheless, GVF effectively mitigates the influence of environmental error through straightforward differential analysis, under the assumption of an unchanging environment, and thereby illustrates the relationship between multidimensional phenotypic traits and genetic variation. Results indicate that the average coefficient of genetic variation for the 9 traits of these 7 offspring is as follows: volume, cardioid area, thickness, largest area, surface area, roundness, width, J index, and length. Except for thickness, 2D and 3D trait values remain larger. This outcome provides stronger evidence that the 3D model better represents and evaluates genetic variation. Finally, the average GVFs corresponding to all traits of 7 varieties were calculated. The ranked GVFs for each variety, from largest to smallest, are *F*_1_, *F*_2_, *F*_7_, *F*_4_, *F*_5_, *F*_3_, *F*_6_. Accordingly, *F*_1_, *F*_2_, and *F*_7_ exhibit superior genetic variations and can be utilized in agricultural breeding hybrid experiments. Thus, the GVF definition could impact breeding processes.

## Discussion

### Establishment of a voxel reconstruction method adapted to seeds

In recent years, 3D reconstruction methods, such as SFM- and MVS-based algorithms, have gained popularity due to their reliance on multi-view feature extraction and matching [40–42]. However, for wheat seed accessions characterized by small size, overall color similarity, and extremely similar texture, these mainstream 3D reconstruction algorithms pose challenges [43,44]. The difficulty in extracting phenotypic features of wheat seeds has led researchers to seek alternative methods, such as employing convolutional neural networks (CNNs) to increase the number of feature points extracted or strengthen the association between a few feature points [45,46]. In pursuing these alternatives, researchers often circumvent the time-consuming and error-prone calibration tasks typically associated with traditional methods.

**Fig. 7.**
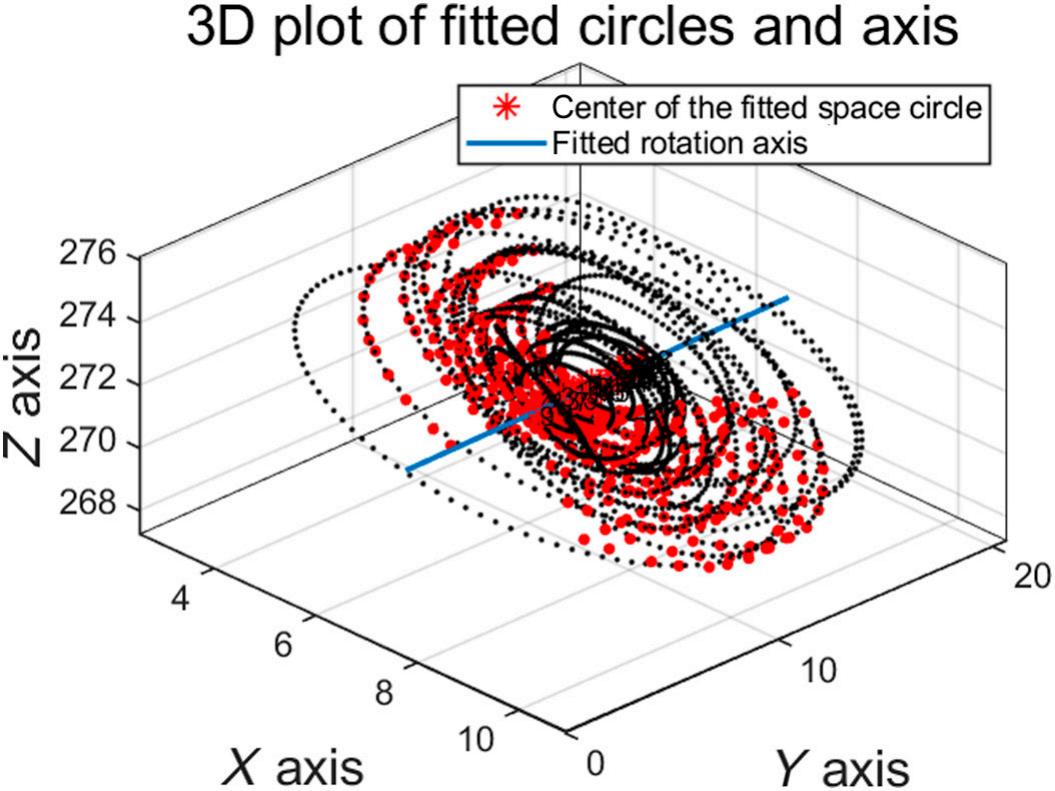
Results of the rotational axis modeling. A total of 28 corner points were randomly selected, and the rotation axis was fitted according to the trajectory of the corner points' rotations.

However, it all became easier when a set of adsorption, rotation, and image capture platforms for wheat seeds was built. With the platform completely fixed, it was only necessary to capture a set of calibration chessboard images, eliminating the need for additional work. A custom rotation model based on platform rotation was designed, ensuring accurate calibration. A critical aspect of the 3D reconstruction process is the fitting of the rotation axis, as illustrated in Fig. 7. The procedure involves 4 steps: (a) generating a rotation trajectory for the same corner, (b) fitting the rotating space circle, (c) calculating the center of the space circle, and (d) fitting the axis of rotation using the center of rotation at various points. This concise and systematic approach ensures accurate alignment of the rotation axis, which is paramount for reliable seed phenotyping and analysis. The smooth morphology and yellow phenotypic color of wheat seeds, which pose challenges for conventional 3D reconstruction algorithms [32], have become the strength of the proposed method. Owing to the completely enclosed morphological characteristics of wheat seeds, the surface can be easily reconstructed with a sophisticated calibration algorithm. This approach overcomes the limitations of existing 3D reconstruction methods and provides a promising alternative for seed phenotyping applications.

### Definition of a new access to gain genetic variation

It is well established that phenotypic variance can be decomposed into genetic variance and environmental error variance. The goal of this study is to obtain a concise formula that captures a factor of genetic variance associated with the presence of parent and offspring while excluding environmental error variance. By applying an engineering treatment of truncation error, the mean phenotypic variance of the 2 parents was calculated and subsequently subtracted from the phenotypic variance of the offspring. The resulting value represents the difference that can be observed from parents to offspring. To eliminate the effects of measurement units, the result was further divided by the mean value of the 2 parents.

Existing parameters that respond to genetic characteristics, such as heritability and combining ability, are available but often require integration with molecular marker techniques for analyzing genotypically distinctive features [47,48]. In order to examine genetic changes more closely from parents to offspring, this study focused on phenotypic data and sought to eliminate the interference of environmental variation by proposing the GVF. One limitation of this approach, however, is the insufficient research on genetics within the scope of this study. This investigation has provided an enlightening pathway for examining genetic variation based on engineering ideas, but further research on genetic correlations is necessary, utilizing the multidimensional phenotypic traits extracted.

### More reasonable approach to genetic variation through diverse traits

Wheat seeds are small, and their phenotypic traits are not easily accessible, resulting in limited measurable traits through manual methods. Traditionally, grain traits were assessed by measuring protein content after crushing, which is a destructive and irreversible measurement. In recent years, valid traits have been obtained through spectroscopic analysis, though these methods entail high costs and require extensive analysis or training of spectral data [49,50]. In contrast, the present study utilizes a set of digital images to model wheat seeds comprehensively and accurately. The low cost and nondestructive nature of this method serve as notable advantages. Furthermore, this approach emphasizes the provision of multidimensional traits that are difficult to obtain via manual measurement. When breeders examine wheat seeds, they may make genetic judgments based on seed head size and ventral groove morphology, which are challenging to measure accurately and easily. This study proposes a method for assessing genetic variation based on multidimensional traits, enabling a more comprehensive and quantitative analysis of wheat seeds using phenotypic traits such as volume, surface area, and the J index.

Undoubtedly, the accuracy of multidimensional traits measurement is directly related to the assessment of genetic variation. Nonetheless, due to the difficulty in measuring phenotypic traits of wheat seeds, the accuracy assessment of the model poses a challenge. In this study, manual measurements of length, width, and thickness were employed to evaluate model precision. Given the substantial error associated with manual measurements, only the *R* value for length (as calipers can easily grip both ends of the wheat) and the RMSE for length, width, and thickness hold referential significance. The results corroborate the feasibility of the 3D reconstruction model. In the future, more precise yet time-consuming and costly 3D measurement methods, such as CT scanning, can be used for accurate evaluation of the multidimensional traits of wheat seeds, thereby serving as a basis for comparison with the model outcomes developed in this study. It is believed that multidimensional traits will play an increasingly prominent role in the assessment of seed genetic variation.

## Conclusion

A reference pathway was presented that uses image-based 3D reconstruction for multidimensional traits measurement and genetic variation assessment with 40 wheat seed image sequences and less than 12 checkerboard images. Rotary axis calibration algorithm was used for seed 3D reconstruction, and 9 traits including length, width, thickness, roundness, surface area, volume, projection area, cardioid area, and J index, were extracted from the 3D model. For accuracy assessment, the correlation coefficient of length is 0.91, and the RMSEs of length, width, and thickness are all less than 0.3 mm. For genetic variation assessment, PCVs among all the traits show significance of multidimensional traits, as seed volume (22.4%), cardioid-derived area (16.97%), and maximum projection area (14.67%). ANOVA shows a highly significance difference among all accessions. The results of GVF innovatively reflect the connection between genotypic variance and phenotypic traits from parents to offspring. These results demonstrate the feasibility of image-based reconstruction for measuring multidimensional traits and assessing genetic variation for wheat seeds without any manual intervention or environmental error. Future studies can apply image-based 3D reconstruction algorithms to genetic variation analysis of other crops and traits, and combine them with genotyping studies to better serve agricultural breeding.

## Data Availability

All data generated or analyzed during this study are available from the corresponding author upon reasonable request. The datasets and code used in this study will be made publicly available in the near future. The location of the data and code will be announced at https://wheatseed.nick163.com.

## Supplementary Materials

Supplementary 1 Extended Tables 1 to 4 Extended Figs. 1 to 4

## References

[1] Jia J, Li H, Zhang X, Li Z, Qiu L. Genomics-based plant germplasm research (GPGR). Crop J. 2017;5(2):166–174.

[2] Whitehead FC. Incorporation of elite subtropical and tropical maize germplasm into elite temperate germplasm. Maydica. 2006;51(1):43–56.

[3] McDonald MB. Seed quality assessment. Seed Sci Res. 1998;8(2):265–275.

[4] Lopes RR, Franke LB, Souza CHL, Bertoncelli P, Graminho LA, Ávila MR, Pereira EA, Motta EAM. Genetic assessment of seed yield-related traits in superior hybrids of Paspalum plicatulum x Paspalum guenoarum. Rev Brasil De Zootec. 2019;48:0075.

[5] Anupama K, Pranathi K, Sundaram RM. Assessment of genetic purity of bulked-seed of rice CMS lines using capillary electrophoresis. Electrophoresis. 2020;41(20):1749–1751. 32357250 10.1002/elps.201900429

[6] Von Wettberg EJB. Ecology and genomics of an important crop wild relative as a prelude to agricultural innovation. Nat Commun. 2018;9(1):649. 29440741 10.1038/s41467-018-02867-z PMC5811434

[7] Smykal P. The impact of genetic changes during crop domestication. Agronomy. 2018;8(7):119.

[8] Deery DM, Jones HG. Field phenomics: Will it enable crop improvement? Plant Phenomics. 2021;2021:9871989. 34549194 10.34133/2021/9871989 PMC8433881

[9] Zhao CJ, Zhang Y, du J, Guo X, Wen W, Gu S, Wang J, Fan J. Crop Phenomics: Current status and perspectives. Front Plant Sci. 2019;10:714. 31214228 10.3389/fpls.2019.00714 PMC6557228

[10] He Q, Tang S, Zhi H, Chen J, Zhang J, Liang H, Alam O, Li H, Zhang H, Xing L, et al. A graph-based genome and pan-genome variation of the model plant Setaria. Nat Genet. 2023;55(7):1232–1242. 37291196 10.1038/s41588-023-01423-w PMC10335933

[11] Varshney RK, Roorkiwal M, Sun S, Bajaj P, Chitikineni A, Thudi M, Singh NP, du X, Upadhyaya HD, Khan AW, et al. A chickpea genetic variation map based on the sequencing of 3,366 genomes. Nature. 2021;599(7886):622–627. 34759320 10.1038/s41586-021-04066-1 PMC8612933

[12] Chen Z, Lancon-Verdier V, le Signor C, She YM, Kang Y, Verdier J. Genome-wide association study identified candidate genes for seed size and seed composition improvement in M. truncatula. Sci Rep. 2021;11(1):4224. 33608604 10.1038/s41598-021-83581-7 PMC7895968

[13] Huang XH, Huang S, Han B, Li J. The integrated genomics of crop domestication and breeding. Cell. 2022;185(15):2828–2839. 35643084 10.1016/j.cell.2022.04.036

[14] Jia H, Li M, Li W, Liu L, Jian Y, Yang Z, Shen X, Ning Q, du Y, Zhao R, et al. A serine/threonine protein kinase encoding gene KERNEL NUMBER PER ROW6 regulates maize grain yield. Nat Commun. 2020;11(1):988. 32080171 10.1038/s41467-020-14746-7 PMC7033126

[15] Yang N, Liu J, Gao Q, Gui S, Chen L, Yang L, Huang J, Deng T, Luo J, He L, et al. Genome assembly of a tropical maize inbred line provides insights into structural variation and crop improvement. Nat Genet. 2019;51(6):1052–1059. 31152161 10.1038/s41588-019-0427-6

[16] Falk K, Jubery TZ, O’Rourke JA, Singh A, Sarkar S, Ganapathysubramanian B, Singh AK. Soybean root system architecture trait study through genotypic, phenotypic, and shape-based clusters. Plant Phenomics. 2020;2020:1925495. 33313543 10.34133/2020/1925495 PMC7706349

[17] Yu B, Boyle K, Zhang W, Robinson SJ, Higgins E, Ehman L, Relf-Eckstein JA, Rakow G, Parkin IAP, Sharpe AG, et al. Multi-trait and multi-environment QTL analysis reveals the impact of seed colour on seed composition traits in Brassica napus. Mol Breed. 2016;36(8):8.

[18] Vafaee Y, Ghaderi N, Khadivi A. Morphological variation and marker-fruit trait associations in a collection of grape (Vitis vinifera L.). Sci Hortic. 2017;225:771–782.

[19] Duan LF, Yang W, Huang C, Liu Q. A novel machine-vision-based facility for the automatic evaluation of yield-related traits in rice. Plant Methods. 2011;7:44. 22152096 10.1186/1746-4811-7-44 PMC3264518

[20] Huang CL, Yang W, Duan L, Jiang N, Chen G, Xiong L, Liu Q. Rice panicle length measuring system based on dual-camera imaging. Comput Electron Agric. 2013;98(3):158–165.

[21] Igathinathane C, Pordesimo LO, Columbus EP, Batchelor WD, Methuku SR. Shape identification and particles size distribution from basic shape parameters using ImageJ. Comput Electron Agric. 2008;63(2):168–182.

[22] Tanabata T, Shibaya T, Hori K, Ebana K, Yano M. High-throughput phenotyping software for measuring seed shape through image analysis. Plant Physiol. 2012;160(4):1871–1880. 23054566 10.1104/pp.112.205120 PMC3510117

[23] Iwata H, Ebana K, Uga Y, Hayashi T, Jannink JL. Genome-wide association study of grain shape variation among Oryza sativa L. germplasms based on elliptic Fourier analysis. Mol Breed. 2010;25(2):203–215.

[24] Li H. Calculation method of surface shape feature of rice seed based on point cloud. Comput Electron Agric. 2017;142:416–423.

[25] Huang X, Zheng S, Gui L, Zhao L, Ma H. Automatic extraction of high-throughput phenotypic information of grain based on point cloud. Trans Chin Soc Agric Mach. 2018;49(4):257–264.

[26] Hu WJ, Zhang C, Jiang Y, Huang C, Liu Q, Xiong L, Yang W, Chen F. Nondestructive 3D image analysis pipeline to extract rice grain traits using x-ray computed tomography. Plant Phenomics. 2020;2020:3414926. 33313550 10.34133/2020/3414926 PMC7706343

[27] Yu LJ, Shi J, Huang C, Duan L, Wu D, Fu D, Wu C, Xiong L, Yang W, Liu Q. An integrated rice panicle phenotyping method based on X-ray and RGB scanning and deep learning. Crop J. 2021;9(1):42–56.

[28] Zhu D, Chen B, Liang X, Yang Y. Apparatus for synchronous measuring three dimensional parameters of maize seeds based on oblique photography. Trans Chin Soc Agric Eng. 2018;34(4):201–208.

[29] Jay S, Rabatel G, Hadoux X, Moura D, Gorretta N. In-field crop row phenotyping from 3D modeling performed using structure from motion. Comput Electron Agric. 2015;110:70–77.

[30] Pound MP, French AP, Fozard JA, Murchie EH, Pridmore TP. Patch-based approach to 3D plant shoot phenotyping. Mach Vis Appl. 2016;27(5):767–779.

[31] Pound MP, French AP, Murchie EH, Pridmore TP. Automated recovery of three-dimensional models of plant shoots from multiple color images. Plant Physiol. 2014;166(4):1688–1698. 25332504 10.1104/pp.114.248971 PMC4256878

[32] Roussel J, Geiger F, Fischbach A, Jahnke S, Scharr H. 3D surface reconstruction of plant seeds by volume carving: Performance and accuracies. Front Plant Sci. 2016;7:745. 27375628 10.3389/fpls.2016.00745 PMC4895124

[33] Matusik W, Buehler C, Raskar R, Gortler SJ, McMillan L. *Image-based visual hulls*. Paper presented at: Proceedings of the 27th Annual Conference on Computer Graphics and Interactive Techniques. New Orleans (LA): SIGGRAPH; 2000;369-374.

[34] Rusu RB, Cousins S. 3D is here: Point Cloud Library (PCL). Paper presented at: IEEE International Conference on Robotics & Automation. Shanghai (China): IEEE.; 2011;1-4.

[35] Duda A, Frese U. Accurate detection and localization of checkerboard corners for calibration. Paper presented at: 29th British Machine Vision Conference. Newcastle (UK): BMVC.; 2018;126.

[36] Lorensen WE, Cline HE. Marching cubes: A high resolution 3D surface construction algorithm. ACM SIGGRAPH Comput Graph. 1987;163–169.

[37] Li YG, Liu X, Ma J, Zhang X, Xu LA. Phenotypic variation in Phoebe bournei populations preserved in the primary distribution area. J For Res. 2018;29(1):35–44.

[38] Ziegler ACD, Tambarussi EV. Classifying coefficients of genetic variation and heritability for Eucalyptus spp. Crop Breed Appl Biotechnol. 2022;22(2):2022.

[39] Hintze JL, Nelson RD. Violin plots: A box plot-density trace synergism. Stat. 1998;52(2):181–184.

[40] Moulon P, Monasse P, Marlet R. others. Global fusion of relative motions for robust, accurate and scalable structure from motion. Paper presented at: IEEE International Conference on Computer Vision. Sydney (Australia): ICCV, 2013;3248-3255.

[41] Schonberger JL, Frahm JM. Structure-from-motion revisited. Paper presented at: IEEE Conference on Computer Vision & Pattern Recognition. Las Vegas (NV): CVPR; 2016;4104–4113.

[42] Magerand L, Del Bue A. Revisiting projective structure from motion: A robust and efficient incremental solution. IEEE Trans Pattern Anal Mach Intell. 2020;42(2):430–443. 29994468 10.1109/TPAMI.2018.2849973

[43] Hafeez J, Lee J, Kwon S, Ha S, Hur G, Lee S. Evaluating feature extraction methods with synthetic noise patterns for image-based modelling of texture-less objects. Remote Sens. 2020;12(23):3886.

[44] Luetzenburg G, Kroon A, Bjork AA. Evaluation of the Apple iPhone 12 Pro LiDAR for an application in geosciences. Sci Rep. 2021;11(1):9. 34782692 10.1038/s41598-021-01763-9 PMC8593014

[45] Jogin M. Feature extraction using convolution neural networks (CNN) and deep learning. Paper presented at: 2018 3rd IEEE International Conference on Recent Trends in Electronics, Information & Communication Technology. Bangalore (India): RTEICT; 2020;2319-2323.

[46] Kashir B, Ragone M, Ramasubramanian A, Yurkiv V, Mashayek F. Application of fully convolutional neural networks for feature extraction in fluid flow. J Vis. 2021;24(4):771–785.

[47] Rasheed A, Xia X, Yan Y, Appels R, Mahmood T, He Z. Wheat seed storage proteins: Advances in molecular genetics, diversity and breeding applications. J Cereal Sci. 2014;60(1):11–24.

[48] Ramesh P, Mallikarjuna G, Sameena S, Kumar A, Gurulakshmi K, Reddy BV, Reddy PCO, Sekhar AC. Advancements in molecular marker technologies and their applications in diversity studies. J Biosci. 2020;45(1):123. 33097680

[49] Zhou L, Zhang C, Taha MF, Wei X, He Y, Qiu Z, Liu Y. Wheat kernel variety identification based on a large near-infrared spectral dataset and a novel deep learning-based feature selection method. Front Plant Sci. 2020;11:575810. 33240294 10.3389/fpls.2020.575810 PMC7683420

[50] Jin S, Zhang W, Yang P, Zheng Y, An J, Zhang Z, Qu P, Pan X. Spatial-spectral feature extraction of hyperspectral images for wheat seed identification. Comput Electr Eng. 2022;101: Article 108077.

