# Supplementary material for: A Pathway to Assess Genetic Variation of Wheat Germplasm by Multidimensional Traits with Digital Images
Source: Plant Phenomics. 2023 Nov 22;5:0119. doi: 10.34133/plantphenomics.0119 (PMC10665127; doi:10.34133/plantphenomics.0119)
Supplement: Supplementary 1 — Extended Tables 1 to 4 Extended Figs. 1 to 4 [file plantphenomics.0119.f1.docx]

**Title**

A Pathway to Assess Genetic Variation of Wheat Germplasm by Multi-dimensional Traits with Digital Images

**Authors**

Tingting Wu^1,2^, Peng Shen^3^, Jianlong Dai^1^, Yuntao Ma^4^, Yi Feng^5,*^

**Affiliations**

1 College of Mechanical and Electronic Engineering, Northwest A&F University, Yangling, Shaanxi 712100, China

2 Key Laboratory of Agricultural Internet of Things, Ministry of Agriculture and Rural Affairs, Yangling, Shaanxi 712100, China

3 College of Information Engineering, Northwest A&F University, Yangling, Shaanxi 712100, China
4 College of Land Science and Technology, China Agricultural University, Beijing 100091, China

5 College of Agronomy, Northwest A&F University, Yangling, Shaanxi 712100, China

^*^Address correspondence to: [fengyiwheat@nwafu.edu.cn](mailto:fengyiwheat@nwafu.edu.cn)

**SUPPLEMENTARY MATERIALS**

Extended Table 1: Relationships of Wheat Seed Varieties Used in the Study

| Variety | Parental Strains | Crossbreeding Batch |
| --- | --- | --- |
| xinong 20 | N/A | N/A |
| xinong 585 | N/A | N/A |
| S16 | xinmai 26, xinong 294 | Batch 1 |
| S18 | xinmai 26, xinong 294 | Batch 1 |
| S19 | xinmai 26, xinong 294 | Batch 1 |
| S20 | xinmai 26, xinong 294 | Batch 1 |
| S26 | xinmai 26, xinong 294 | Batch 1 |
| S28 | xinmai 26, xinong 294 | Batch 1 |
| S76 | xinong 20/02Ta, xinong 585 | Batch 2 |
| S77 | xinong 20/02Ta, xinong 585 | Batch 2 |
| S78 | xinong 20/02Ta, xinong 585 | Batch 2 |
| S79 | xinong 20/02Ta, xinong 585 | Batch 2 |
| S80 | xinong 20/02Ta, xinong 585 | Batch 2 |
| S81 | xinong 20/02Ta, xinong 585 | Batch 2 |
| S82 | xinong 20/02Ta, xinong 585 | Batch 2 |

Extended Table 2: Camera Calibration Error Assessment

| Image Number | Average Error per Pixel |
| --- | --- |
| Image 1 | 0.0596758 |
| Image 2 | 0.0417707 |
| Image 3 | 0.0401347 |
| Image 4 | 0.019331 |
| Image 5 | 0.0170522 |
| Image 6 | 0.0343147 |
| Image 7 | 0.0504686 |
| Image 8 | 0.0646733 |
| Image 9 | 0.0469557 |
| Image 10 | 0.0896334 |
| Overall Average Error | 0.046401 |

Note: Calibration was conducted using 10 images of a calibration board from different angles. The camera's intrinsic parameters obtained from the calibration were then back-projected onto the image. The error is calculated based on the pixel difference between the projected points and the original corners of the calibration board.

Extended Table 3: Differences between Volume Value of CT and Our Reconstruction

| Variety of wheat seed | Number of the variety | Volume of CT results | Volume of our results | Absolute Error$({mm}^{3})$ | Relative Error |
| --- | --- | --- | --- | --- | --- |
| YL20 | H1 | 26.539 | 24.7195 | 1.8195 | 0.0736 |
| YL20 | H2 | 37.549 | 36.0496 | 1.4994 | 0.0415 |
| YL20 | H3 | 26.613 | 25.4795 | 1.1335 | 0.0444 |
| YL20 | H4 | 24.92 | 24.2501 | 0.6699 | 0.0276 |
| YL20 | H5 | 26.987 | 27.0014 | 0.0144 | 0.0005 |
| YL20 | H8 | 29.289 | 29.4415 | 0.1525 | 0.0051 |
| YL20 | H9 | 26.406 | 26.3514 | 0.0545 | 0.0020 |
| YL20 | H10 | 32.455 | 32.9336 | 0.4786 | 0.0145 |
| XN585 | D1 | 36.549 | 36.1349 | 0.4140 | 0.0114 |
| XN585 | D2 | 33.177 | 31.2291 | 1.9479 | 0.0623 |
| XN585 | D3 | 37.989 | 33.4348 | 4.5541 | 0.1362 |
| XN585 | D4 | 23.782 | 21.815 | 1.9670 | 0.0901 |
| XN585 | D5 | 39.378 | 36.9524 | 2.4256 | 0.0656 |
| XN585 | D6 | 33.864 | 31.9515 | 1.9125 | 0.0598 |
| XN585 | D7 | 44.117 | 41.947 | 2.1699 | 0.0517 |
| XN585 | D8 | 38.262 | 34.9603 | 3.3017 | 0.0944 |
| XN585 | D9 | 39.08 | 36.3721 | 2.7079 | 0.0744 |
| XN585 | D10 | 33.891 | 31.6816 | 2.2094 | 0.0697 |
| MEAN | / | / | / | 1.63515 | 0.0514 |

Note: Eighteen seeds were randomly selected for CT reconstruction and reconstruction using our method. The unit for volume results is ${mm}^{3}$. Finally, the relative error and the average error were calculated.

Extended Table 4: Heritability calculation results for 9 traits

| Trait | Heritability |
| --- | --- |
| Length | 59.6% |
| Width | 58.5% |
| Height | 52.7% |
| Surface Area | 71.8% |
| Volume | 74.9% |
| Projection Area | 65.6% |
| Roundness | 11.0% |
| Cadioid Area | 70.3% |
| J Index | 37.3% |

Note: The heritability calculation model used was based on single-location data across two years using only phenotypic traits. The model assumes that the total phenotypic variance is the sum of genetic and environmental variances, and it provides a measure of how much of the observed variation in phenotype can be attributed to genetic differences among individuals.

Extended Figure 1: Extended Figure 1: Checkerboard images from different viewpoints. Within the visual platform, only an initial calibration with 10 checkerboard images is needed to determine the axis of rotation. Subsequent experiments do not require any further calibration.

Extended Figure 2: Wheat seed images from various viewpoints. During actual photography, images are generally taken every 9 or 10 degrees, resulting in a total of 40 or 36 wheat seed images, respectively. Here, we have selected the first 20 images from a complete 40-image sequence.

|  |  |
| --- | --- |

Extended Figure 3: Comparison of width and thickness. While length can be easily measured, the width and thickness measurements exhibit larger discrepancies due to the random error introduced during manual assessment. This error is greater than the true value differences among various varieties, which leads to the fewer $R^{2}$. However, RMSE of the 3 traits were within 0.3.

Extended Figure 4: A comparison between the results of the voxel reconstruction using the space carving method and the results presented in this paper. The former exhibits more evident cut marks on the surface, with the wheat seed groove area not being distinctly represented. For the top part of the wheat seed, the former displays significant slope variations. In contrast, our method reconstructs a smoother wheat seed surface, aligning more closely with the actual model.
